# Supplementary material for: Coordination of multiple joints increases bilateral connectivity with ipsilateral sensorimotor cortices
Source: Neuroimage. Author manuscript; Available in PMC 2020 Apr 30. (PMC7192312; doi:10.1016/j.neuroimage.2019.116344)
Supplement: SupplementaryFig&Table [file NIHMS1580098-supplement-SupplementaryFig_Table.docx]

Supplementary Figure 1. Models tested for DCM analysis. Models 1-6 allow for only linear intrinsic connections (dashed lines within regions) and both nonlinear and linear extrinsic connections (solid lines between regions), while Models 7-12 allow both nonlinear and linear intrinsic and extrinsic connections (all solid lines). Individual models differ in interhemispheric connections allowed between M1 and PM regions. Connections missing from each model are highlighted in red. The left side of each model is the contralateral side.

Supplementary Figure 2. A representative example of the observed and model-simulated spectrograms for each region for one participant using the winning model (Model 12) averaged across trials. (A-Top) The observed spectrogram for Hand Opening (HO). (A-Bottom) The model-simulated spectrogram for HO. (B-Top) The observed spectrogram for Hand Opening while Lifting (HOL). (B-Bottom) The model-simulated spectrogram for HOL. Red represents an increase in power compared to baseline and blue represents a decrease in power compared to baseline. 0 ms indicates movement onset. Overall, the model explained ~80% of the original spectral variance for each condition.

Supplementary Figure 3. Default Oscillatory Coupling for Lifting only in a small cohort of participants (N=3). Arrows indicate directional connections showing coupling with the motor network. The color of the arrow indicates the frequency band involved. Arrows that change colors represent cross-frequency coupling. Solid lines indicate positive coupling while dashed lines indicate negative coupling. Contra = Contralateral hemisphere; Ipsi = Ipsilateral hemisphere.

**Supplementary Table 1. Exceedance probabilities for each family**

| Model Family | Open | Lift + Open |
| --- | --- | --- |
| Linear | 0.0001 | 0.0002 |
| Nonlinear | 0.9999 | 0.9998 |

**Supplementary Table 2. Exceedance probabilities for each model**

| Models | Open | Lift + Open |
| --- | --- | --- |
| 7 | 0.0001 | 0.0008 |
| 8 | 0.0001 | 0.0001 |
| 9 | 0.0003 | 0.0003 |
| 10 | 0.0000 | 0.0005 |
| 11 | 0.0001 | 0.0002 |
| 12 | 0.9994 | 0.9981 |
